# The impact of the nodal status on the overall survival of non-surgical patients with esophageal squamous cell carcinoma

**DOI:** 10.1186/s13014-019-1365-2

**Published:** 2019-09-03

**Authors:** Zongxing Zhao, Yanan Zhang, Peiliang Wang, Xin Wang, Minghuan Li

**Affiliations:** 1 School of Medicine, Shandong University, Jinan, Shandong China; 2 Department of Radiation Oncology, Shandong Cancer Hospital and Institute, Jinan, Shandong China; 3 Department of Radiation Oncology, Liaocheng People’s Hospital, Liaocheng, Shandong China; 4 Department of Health Care, Liaocheng People’s Hospital, Liaocheng, Shandong China; 5 Shandong First Medical University & Shandong Academy of Medical Sciences, Jinan, Shandong China

**Keywords:** Esophageal squamous cell cancer, Non-surgical, Metastatic lymph nodes, Definitive radiotherapy, Prognosis

## Abstract

**Background:**

The prognosis of N categories for patients with non-surgical esophageal carcinoma based on the number of metastatic lymph nodes is controversial. The present study analyzes prognostic implications of the number, extent, and size of metastatic lymph nodes for patients with esophageal squamous cell carcinoma (ESCC) treated with definitive (chemo-)radiotherapy to provide more information on treatment strategy.

**Methods:**

We reviewed 357 ESCC patients treated with definitive radiotherapy between January 2013 and March 2016 retrospectively. We assessed potential associations between the involved extent (N0, 1 region, 2 regions, and 3 regions), number (N0, 1–2, 3–6, and ≥ 7), and size (N0, ≤2 cm, and > 2 cm) of metastatic lymph nodes and overall survival. Multivariate analyses of the clinicopathological factors were performed using the Cox proportional hazard model.

**Results:**

5-year survival rates were 43.6% for patients in the N0 group and 29.3% in the N+ group (*p* = 0.001). Kaplan-Meier analyses for all cases revealed that there were significant differences in survival based on the extent (the OS rates at 3 years were 53.3% for patients in the N0 group, 45.7% in the 1 region-involved group, 28.0% in the 2 regions-involved group, and 13.3% in the 3 regions-involved group, *P* < 0.001), number (the OS rates at 3 years were 49.0% for patients in the 1–2 LNs group, 27.8% in the 3–6 LNs group, 0 in the ≥7LNs group, *P* < 0.001), and size (the OS rates at 3 years were 41.6% for patients in the LNs ≤2 cm group and 20.7% in the LNs > 2 cm group, *P* = 0.001) of metastatic LNs. One hundred seventy-two patients (48.2%) had experienced GTV failure, 157 (43.1%) had distant failure, 49 (13.7%) had out-of-GTV nodal failure, and 70 patients (19.6%) had no evidence of disease at the last follow-up. Nodal status correlated statistically with GTV failure. Patients with LN metastases in the abdominal region had worse survival rates than those with metastases in the other regions. The extent and number of metastatic LNs, T category, Primary tumor location, and chemotherapy were independent prognostic factors of OS in multivariate analyses.

**Conclusions:**

For patients with ESCC who received definitive (chemo-)radiotherapy, the number, extent, and size of metastatic LNs were prognostic factors, particularly of the T2/3 disease. Patients with LN metastases in the abdominal region had worse survival.

## Background

Esophageal carcinoma (EC) is a highly lethal disease. Of the two predominant histologic types, squamous cell carcinoma is the most common histological type in China, where it accounts for more than 90% of esophageal carcinoma cases [1]. Radiotherapy has been established as a definitive treatment for unresectable or medically inoperable tumors in ESCC patients. Chemotherapy has been added to the treatment and serves two purposes, including radiosensitization and control of micrometastatic diseases. Several randomized trials have demonstrated local control and survival benefit from chemoradiotherapy in patients with ESCC. There is advocacy for definitive chemoradiotherapy (dCRT) to serve as an alternative treatment for EC [2, 3]. Lymph node metastases are the most important factors affecting esophageal cancer prognosis [4, 5]. However, increasing numbers of reports are showing that the N-classification based on the number of metastatic lymph nodes (LNs) remains imperfect [6]. Some studies have shown that the extent of metastatic LNs could have significant prognostic implications for survival [7, 8].

Locoregional recurrence is reportedly more common after dCRT than after surgery [9]. However, there is little available information on how the status of metastatic lymph nodes influences prognosis for non-surgical patients with ESCC. In this study, we analyzed the prognostic implications of metastatic lymph nodes, including the factors of number, extent, and size, on the overall survival of ESCC patients who received definitive (chemo)radiotherapy.

## Material and methods

### Patients

Between January 2013 and March 2016, 357 patients with histologically confirmed ESCC received definitive radiotherapy or chemoradiotherapy at Shandong Cancer Hospital Affiliated to Shandong University. The treatment strategies were made by a multidisciplinary team, including radiation oncologists, oncologists, and surgeons dedicated to thoracic malignancies. All patients underwent pretreatment staging workups, including tumor biopsy, esophagoscope, esophageal ultrasound (EUS), barium swallow, chest and abdominal computed tomography (CT), and/or fluorodeoxyglucose-positron emission tomography (FDG-PET) scan. A positive LN was defined by a short-axis length greater than 1 cm on CT or by a short-axis diameter of the paraesophageal, tracheoesophageal sulcus, pericardial angle, or abdominal LN greater than 5 mm [10]. LNs were considered to be positive when at least one of the following criteria was met: size ≥10 mm, round shape, hypoechoic pattern or clearly visible borders using EUS, or when the maximum standard uptake value (SUVmax) was higher than the background blood pool activity measured in the thoracic aorta or normal liver parenchymal activity. Patients were classified based on the extent (cervical, thoracic, and abdomen) of metastatic LNs (N0, 1 region, 2 regions, and 3 regions) according to the 8th edition of the AJCC staging system for ESCC. We only included patients with the T2, T3, and T4 diseases because the number of Tis-T1 that received radical radiotherapy or chemoradiotherapy was too small to be studied further. This study was approved by the medical ethics committees of the Shandong Cancer Hospital Affiliated to Shandong University, and informed consent was waived.

### Treatment

A planned computed tomography scan was performed using an intravenous contrast with a slice thickness of 3 mm. Each patient’s images were transported to the treatment planning system to design the plan. FDG-PET was imported into the Eclipse Planning System (Varian Medical System, Palo Alto, CA) if needed. Radiotherapy was delivered using a dynamic multi-leaf linear accelerator with photon energies of 6 MV. The gross tumor volume (GTV) was defined as the visible macroscopic tumor and positive LNs based on all available clinical and imaging data. The clinical target volume (CTV) comprised the cranial and caudal margins of 3–5 cm and radial margins of 0.8–1.0 cm of a primary tumor and regional lymph node area at risk of microscopic disease. The planning target volumes were defined by 0.5–0.8 cm expansions of the CTV. Planning target volume (PTV) was delivered at a total dose of up to 50.4–66 Gy in 28–33 fractions (5 days a week, 5 to 6 weeks). Two hundred and twenty-seven patients received concurrent platinum-based chemotherapy with 5-fluorouracil or paclitaxel.

### Follow up

Patients were followed-up every 3 months after treatment for the first 2 years, every 6 months for the next 3 years, and every 12 months after that. All patients underwent physical examinations, including barium swallow, cervical, chest, and abdominal enhanced CT scans, and endoscopy to assess recurrence or metastasis. When necessary, FDG-PET was performed in response to specific symptoms. We assessed failure patterns on post-treatment images, including esophagogram, endoscopy, CT, or PET/CT scans. Patterns of failure were defined based on the sites of failure and included GTV (primary tumor and original LN) failure, out of GTV LN failure, and distant failure. The last general follow-up of survivors was done in January 2019. Overall survival (OS) was defined as the time from the date of pathologic diagnosis to the date of death. Surviving patients were censored on the day of the last contact.

### Statistical analysis

Survival proportions were estimated using the Kaplan-Meier method. Multivariate and the univariate analyses were performed separately using the Cox proportional hazard model and log-rank test. A *p*-value of less than 0.05 was considered statistically significant. All statistical analyses were carried out using SPSS 23.0 (SPSS, Chicago, IL, USA).

## Results

### Clinical characteristics of the population and LNs

Complete data were available for 357 patients. The median follow-up was 27.8 months (range, 2–74 months), and the median age was 62 years (range, 43–83 years). Two hundred and twenty-seven of the 357 patients underwent chemoradiotherapy. Two hundred and sixty-three of the total patients had LN metastasis, with the median number of the involved LNs being 2 (range, 1–12). The median size of the involved LNs was 1.1 cm (range, 0.5–5.1 cm). Of the 357 patients, the 1 region-involved (48.2%, 172 of 357) was the most common, followed by N0 (26.3%, 94 of 357), 2 regions-involved (21.3%, 76 of 357), and 3 regions-involved (4.2%, 15 of 357). The detailed information is summarized in Table 1. The size of LNs correlated statistically with the T category, the number of LNs, and the extent of LNs (*P* < 0.001).

**Table 1:** Patient characteristics and univariate analysis of prognostic factors

Variables	Number ofpatients (%)	Mediansurvival (m)	3-y survivalrate (%)	*P*
Age (years)				0.248
<60	105 (29.4%)	34.0	47.8%	
≥ 60	252 (70.6%)	27.8	40.3%	
Sex				0.377
Male	282 (79.0%)	28.0	41.1%	
Female	75 (21.0%)	32.6	48.3%	
Primary tumor location				<.001
Upper	168 (47.1%)	42.0	55.7%	
Middle	132 (37.0%)	26.0	35.4%	
Lower	57 (15.9%)	16.0	21.1%	
T category				<.001
T2	76 (21.3%)	49.0	59.2%	
T3	217 (60.8%)	29.0	42.2%	
T4	64 (17.9%)	17.5	24.3%	
No. of LNs				<.001
0	94 (26.3%)	46.0	53.3%	
1–2	150 (42.0%)	36.0	49.0%	
3–6	96 (26.9%)	18.0	27.8%	
≥ 7	17 (4.8%)	13.0	0	
Extent of LNs				<.001
0	94 (26.3%)	46.0	53.3%	
1 region	172 (48.2%)	31.0	45.7%	
2 regions	76 (21.3%)	19.0	28.0%	
3 regions	15 (4.2%)	12.0	13.3%	
Size of LNs				0.001
0	94 (26.3%)	46.0	53.3%	
≤ 2 cm	220 (61.6%)	28.0	41.6%	
>2 cm	43 (12.1%)	17.0	20.7%	
Treatment				0.010
CRT	227 (63.6%)	33.8	47.6%	
RT alone	130 (36.4%)	25.6	33.7%	

*LNs* Lymph nodes, *CRT* Chemoradiotherapy, *RT* Chemoradiotherapy

### Survival

**Fig. 1:**
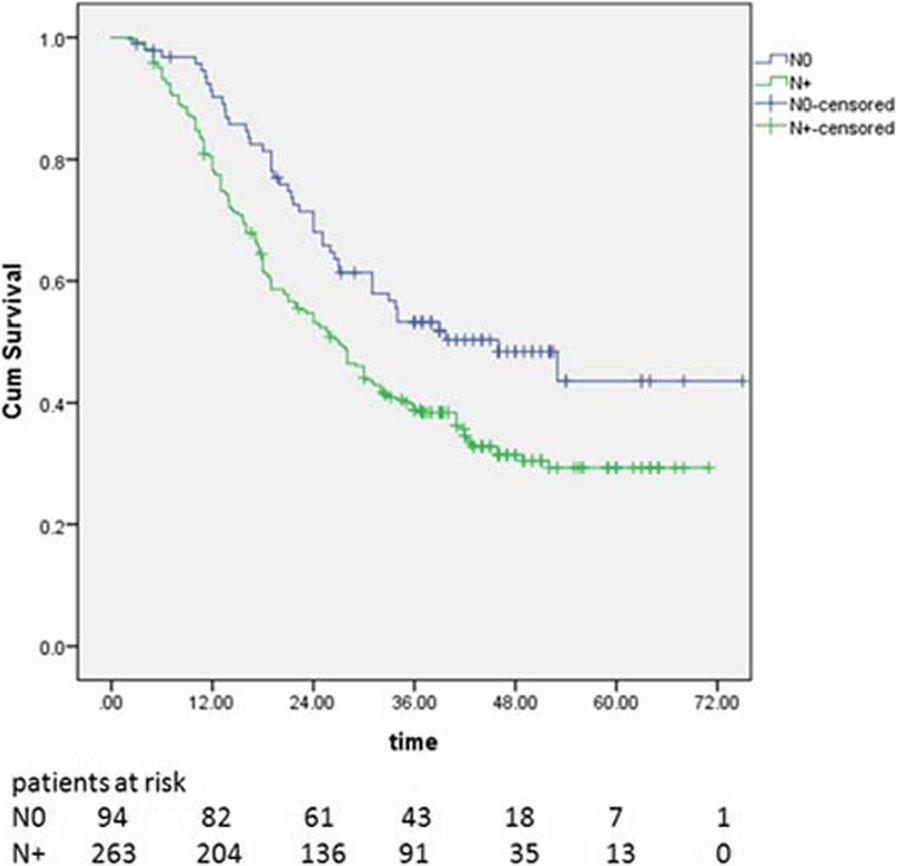
Kaplan- Meier survival curves for patients based on the status of LNs

The 1-, 3-, and 5-year OS rates were 81.3, 42.6, and 33.2%, respectively. The median survival time was 30.5 months for all patients. Survival rates for the N0 group were 90.2% at 1 year, 53.3% at 3 years, and 43.6% at 5 years, and the Median survival time was 46.0 months. Survival rates for the N+ group were 78.2% at 1 year, 38.8% at 3 years, and 29.3% at 5 years, and the Median survival time was 27.1 months. Survival rates in the N0 group were significantly better than those in the N+ group (*p* = 0.001 Fig. 1).

At the time of the last follow-up contact, 172 patients (48.2%) had experienced GTV failure, 157 (43.1%) had distant failure, 49 (13.7%) had out-of-GTV nodal failure, and 70 patients (19.6%) had no evidence of disease. The risk of GTV failure by the different nodal size groups is shown in Table 2. After adjusting age, sex, treatment, and T-category according to the 8th AJCC system, multivariate analyses showed that there were significant differences in hazard ratios (HRs) for GTV failure between the 3–6 LNs group (HR, 2.445), ≥7LNs group (HR, 2.457), 2 regions-involved group (HR, 2.189), 3 regions-involved group (HR, 3.081), LNs > 2 cm group (HR, 2.237), and N0 group (HR, 1). However, the HRs of other groups were similar to those of patients with N0 (HR, 1).

**Table 2:** Effect of nodal status on risk of GTV failure

Variables	Hazard Ratio	95% CI	*P*
No. of LNs (baseline, N0)			0.001
1–2	1.025	0.684–1.538	0.903
3–6	2.445	1.627–3.672	0.012
≥ 7	2.457	1.222–4.941	0.001
Extent of LNs (baseline, N0)			0.001
1 region	1.135	0.771–1.672	0.521
2 regions	2.189	1.434–3.340	0.001
3 regions	3.081	1.482–6.406	0.002
Size of LNs (baseline, N0)			0.006
≤ 2 cm	1.331	0.922–1.921	0.127
>2 cm	2.237	1.367–3.661	0.001

*GTV* Gross tumor volume, *CI* Confidence interval

The Kaplan-Meier analyses for OS show that there were significant differences in survival based on the lymph node involved extent (N0, 1 region, 2 regions, and 3 regions, *P* < 0.001), number (N0, 1–2, 3–6, and ≥ 7, *P* < 0.001), and size (N0, ≤2 cm, and > 2 cm, *P* = 0.001). For 263 patients with metastatic LNs, the OS rates at 3 years were 49.0% for the 1–2 LNs group, 27.8% for the 3–6 LNs group and 0 for the ≥7LNs group based on the number, with corresponding median survival times of 36.0, 18.0 and 13.0 months, respectively. The OS rates at 3 years were 45.7% for the 1 region-involved group, 28.0% for the 2 regions-involved group and 13.3% for the3 regions-involved group based on the extent, with corresponding median survival times of 31.0, 19.0 and 12.0 months, respectively. The OS rates at 3 years were 41.6% for the LNs ≤2 cm group and 20.7% for the LNs >2 cm group based on the size, with corresponding median survival times of 28.0 and 17.0 months, respectively.

Table 3 lists the incidences of metastasis in different regions. The 3-year survival rates of patients with cervical region-involved, a thoracic region-involved only, and abdominal region-involved were 38.2, 43.4, and 16.2%, respectively, and their median survival times were 28.0, 28.5, and 14.5 months, respectively. The Kaplan-Meier analyses show that there were significant differences in survival according to the different involved regions (*P* < 0.001 Table 3).

**Table 3:** Overall survival of 357 patients based on the LNs Location

	No. ofPatients (%)	MedianSurvival (m)	3-YearSurvival (%)	*P* Value
N0	94 (26.3%)	46	53.3%	< 0.001
Cervical region-involved	73 (20.4%)	28.0	38.2%	
Thoracic region-only	156 (43.7%)	28.5	43.4%	
Abdominal region-involved	51 (14.3%)	14.5	16.2%	

17 patients had both Cervical and Abdominal region-involved LNs

*LNs* Lymph nodes

**Fig. 2:**
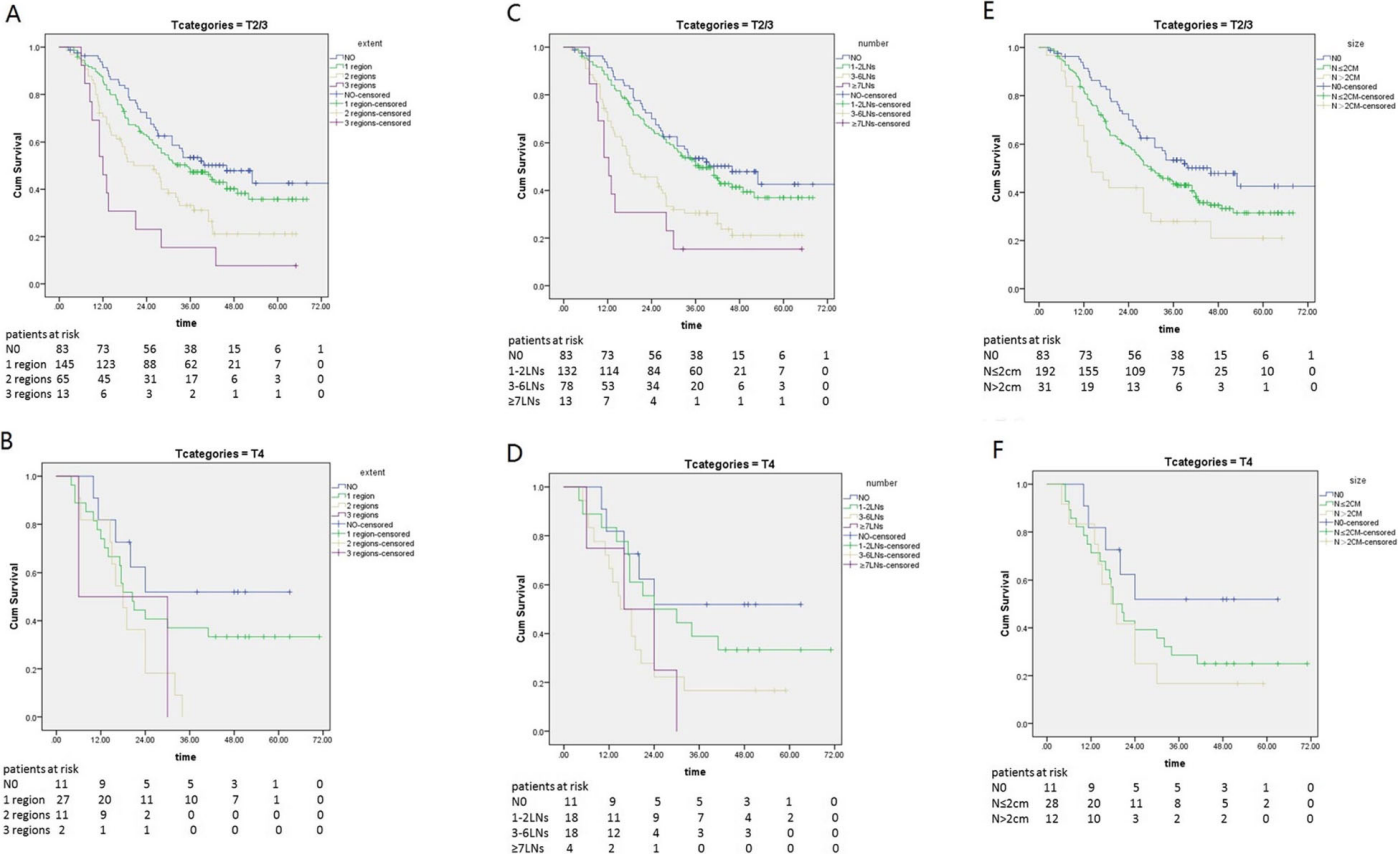
Kaplan- Meier survival curves for patients based on the extent of metastatic LNs in the T2/3 subgroup (**a**) and the T4 subgroup (**b**) (*p* = 0.128), the number of metastatic LNs in the T2/3 subgroup (**c**) and the T4 subgroup (**d**) (*p* = 0.119), the size of metastatic LNs in the T2/3 subgroup (**e**) and the T4 subgroup (**f**) (*p* = 0.128)

We performed analyses on patients in the T2/3 or T4 subgroups to explore the statuses of the LNs in predicting survival in different T categories. There were significant differences in survival in the T2/3 subgroup between patients based on the extent (*P* < 0.001 Fig. 2a), number (*P* < 0.001 Fig. 2c), and size (*P* = 0.001 Fig. 2e) of metastatic LNs. However, there were no significant differences between patients within the T4 stage (Fig. 2b, d, f) based on these factors.

### Univariate and multivariate analyses of prognostic factors

Univariate survival analyses showed that the T category, Primary tumor location, chemotherapy, and statuses of metastasis LNs were significantly associated with OS (Table 1). Table 4 presents the results of the multivariate analyses for OS. In the current study, the extent and number of metastatic LNs, T category, Primary tumor location, and concurrent chemotherapy were independent prognostic factors.

**Table 4:** Multivariate Cox regression analysis of the prognostic factors for OS in patients with ESCC

Prognostic Factor	HazardRatio	95% CI	*P*
Age(<60 vs ≥60)	0.890	0.637–1.243	0.495
Sex (Male vs Female)	0.722	0.503–1.304	0.076
Primary tumor location (baseline, Upper)			0.010
Middle	1.292	0.948–1.761	0.105
Lower	1.790	1.229–2.607	0.002
T category (baseline, T2)			0.013
T3	1.171	0.795–1.724	0.425
T4	1.827	1.162–2.872	0.009
No. of LNs (N0, < 3 vs ≥3)	1.667	1.177–2.361	0.004
Extent of LNs (baseline, N0)			0.044
1 region	1.365	0.834–2.087	0.125
2 regions	1.575	0.989–2.508	0.056
3 regions	2.475	1.178–5.159	0.016
Size of LNs (N0, ≤2 vs > 2 cm)	0.812	0.527–1.252	0.347
Treatment (CRT vs RT alone)	0.569	0.425–0.762	0.001

*LNs* Lymph nodes, *CI* Confidence interval, *CRT* Chemoradiotherapy, *RT* Radiotherapy

## Discussion

The American Joint Committee on Cancer (AJCC) tumor node metastasis (TNM) cancer staging system has been used widely to guide treatment decision and evaluate prognosis. The 8th edition staging of esophageal carcinoma presents separate classifications for clinical (cTNM), pathologic (pTNM), and post-neoadjuvant (ypTNM) stage groups. Some studies suggest that both the number of metastatic LN station and the ratio of metastatic LN number to total harvested LN number are important prognostic factors. Mariette C et al. found that 5-year survival rates were significantly poorer for patients with a ratio of metastatic LNs (LNR) > 0.2 than survival rates for patients with a ratio of metastatic LNs (LNR) ≤0.2 (22% vs. 54%, *P* < 0.001) [11]. 5-year survival rates in another study were 30, 16, and 13% for patients with LNR ≤0.2, 0.21–0.5, and > 0.5, respectively (*P* < 0.001) [12]. However, these data provide little references to patients who received non-surgical treatment.

Clinical nodal staging is now also based on the number of metastatic LNs. Endosonographic-directed fine needle aspiration (EUS-FNA) is strongly recommended by the AJCC to confirm the histologic diagnosis of LNs for accurate clinical staging [13]. EUS-FNA is invasive and impracticable for patients with multiple LNs and is, therefore, not widely used in clinical practice. The FNA of peritumoral lymph nodes should be avoided because the needle would have to pass through the primary tumor in the esophageal wall, which could lead to a false-positive result [14]. EUS, CT, and FDG-PET afford regional lymph node imaging and are the principal non-invasive pretreatment staging workups. EUS is reportedly the most sensitive method for the detection of regional lymph node metastases, whereas CT and FDG-PET have higher specificity [15]. Each imaging modality has its advantages and disadvantages; hence, CT, EUS, and PET should be considered complementary diagnostic methods. A recent study showed that the accuracy for N staging was 66% for EUS, 68% for PET, and 63% for CT [16], which is probably one of the reasons why prognostic implications for clinical categories will not be equivalent to those of pathologic categories. It is certainly worth analyzing the prognostic implications of metastatic LNs by using the information on their radiological features.

Our study indicates that the status of metastatic LNs, including the number, extent, and size, are prognostic factors for patients with ESCC who received radical radiotherapy. Per univariate analyses (*P* < 0.001), there were significant differences in survival based on the number of metastatic LNs according to the 8th edition of the AJCC staging system. However, further subgroup analyses showed that survival in N2 and N3 patients did not differ (*p* = 0.181). Several previous studies that focused on operable ESCC also reported similar results. For example, Yamasaki et al. [17] and Chen et al. [18] recently revealed that there was no significant difference in survival between the N2 and N3 categories according to the AJCC TNM staging system in surgical patients. We also found that N0 versus N1 showed no significant difference in the survival of all cohort patients (*p* = 0.280). According to data for clinically staged patients from the Worldwide Esophageal Cancer Collaboration (WECC) by T W Rice et al., of 8156 clinically staged patients with squamous cell carcinoma, Non-risk-adjusted survival for ESCC was not distinctive for cN0 versus cN1 [6]. It is, therefore, necessary to determine the optimal cutoff points for the number of metastatic LN for prognosis in non-surgical patients. Several studies, for example, have reported significant differences in prognosis between patients with different metastatic lymph nodes (N0, 1–3, ≥4) [11, 19, 20]. However, we found that significant differences in survival were observed overall and within each subgroup when patients were classified based on the extent of metastatic LNs involvement (N0, 1 region, 2 regions, and 3 regions). More emerging data are consistent with this result. Peng J et al. [7] and Ning et al. [8] proposed modified nodal categories based on the number of metastatic LN stations for staging ESCC to discriminate the survival differences among groups better.

No previous evidence is available to prove that the size of metastatic LNs is of prognostic significance to OS or DFS. Our data showed that the size of the lymph node was significantly interrelated with extent and number. Therefore, the significant effect of size in predicting survival seen in univariate, but not in multivariate analyses, could be accounted for by the influence from extent and number. Also, the size of an LN was significantly interrelated with extranodal neoplastic spread (ENS). A previous report found ENS to be present in 27.2% of patients with nodes measuring < 2 cm, 55.8% of those with nodes measuring more than 3 to 4 cm, and 100% of those with nodes measuring > 5 cm in head and neck cancers [21]. Because of the effect of ENS on prognosis and treatment [22], the size of a lymph node was still retained in the AJCC staging systems for head and neck carcinoma. In the present study, there were fewer patients with involved LNs greater than 2 cm, which may have also affected the results of the analysis.

In our study, 172 patients (48.2%) had experienced GTV failure, 157 (43.1%) had distant failure, and 49 (13.7%) had out-of-GTV nodal failure. Similar results were observed in a previous study by Zhang et al. who retrospectively assessed patients with locally advanced ESCC who had received IFI. With a median follow-up of 52.6 months, primary lesion and involved regional LN failure, distant metastasis, and initially, uninvolved LN failure were seen in 53.75, 41.25, and 30% of patients, respectively. The authors also found that there were no significant differences in OS for patients with and without initially, uninvolved LN failure [23]. The main pattern of regional recurrence was GTV failure in those with advanced-stage ESCC. Patients with > 6 nodes, 3 regions-involved, and nodes > 2 cm in our study had a poor prognosis. We also found that the HRs for a GTV failure in these patients were higher than those in other groups. The OS rates at 3 years were 0 for the ≥7LNs group, 13.3% for the 3 regions-involved group, and 20.7% for the LNs > 2 cm group; the vast majority of patients were not curable. However, our study is retrospective. Further work needs to be done to establish whether non-surgical treatment should be delivered with curative intent to these patients.

For locally advanced ESCC, the primary tumor is the most important factor affecting survival. Li et al. retrospectively evaluated the failure patterns of 56 patients with clinical T4 M0 and found that 48 patients (85.7%) had experienced failure: 39 (69.6%) in-field, 7 (12.5%) elective nodal, and 19 (33.9%) distant, with only 1 patient (1.8%) experiencing isolated elective nodal failure. The authors also found that there was no significant difference in the median OS of the patients with and without regional lymph node metastases (8 months vs. 7 months; *p* = 0.898) [24]. For T4 stage, the predominant pattern after definitive CRT is a local failure, which is associated with worse overall survival [25, 26]. The high local recurrence rate could have masked regional nodal failure because most of the patients died before their regional nodal failure. Perhaps this is the reason why the regional LN failure was not the main pattern of recurrence in these advanced stage ESCC patients. In the current study, we found that the nodal status, including number, extent, and size, had no significant influence on survival among patients with T4 stage, suggesting that we should pay more attention to primary tumors for patients with the T4 disease. When we develop treatment strategies, a limited radiation therapy target volume, such as Involved-field RT, rather than elective nodal irradiation, should be performed to minimize toxicity.

EC with abdominal nodal metastases is a strong predictor of poor outcome [27, 28]. In the 6th edition of the AJCC staging system, celiac LNs are defined as M1a stage in the lower third esophageal cancer and as M1b stage in the upper and middle third esophageal cancer [29]. However, the 7th AJCC staging system redefines celiac LNs as regional LNs [30]. This modification has proven to be controversial. Rutegard M et al. retrospectively analyzed 446 patients with distal esophageal cancer after resection and found that, compared to celiac node-negative patients, celiac node-positive patients were at a 52% increased risk of disease-specific mortality [31]. The authors’ view was that patients with distal esophageal cancer with celiac node metastasis seem to have similar poor survival to patients with more distant metastasis. For patients with celiac LNs metastasis receiving CRT, the celiac LNs metastasis group was found to have worse PFS and OS than the non-celiac LNs metastasis group [32]. In our study, the abdominal region included the station of paracardial, left gastric, common hepatic, splenic, and celiac nodes. The 3-year overall survival was 16.2% in the abdominal region involved, which had worse survival rates than those with metastases in the other regions. The prognostic value of abdominal LNs metastases, especially of celiac nodes, needs to be confirmed in large sample size, and it should be considered as a subcategory in staging systems.

There are some limitations to this study. First, our research was a retrospective study at a single institution, and the number of patients was relatively small, especially in the T1 and T4 subgroups. We need bigger data, including T stage, length, and multi-dimensional lymph node status to define the clinical staging of esophageal cancer, guide its treatment, and predict its prognosis. Secondly, we did not analyze the influence of histologic differentiation on prognosis because it would be obtained with difficulty in most patients confirmed by biopsy.

## Conclusions

Apart from the primary tumor, we think that the LNs status, including number, extent, and size, should also be taken into account for prognostic implications for non-surgical patients with ESCC, rather than utilizing only one of them. These findings also provide further guidance for treatment strategy and the future staging system of EC. For selected patients with poor prognoses, such as those with ≥7LNs, further research needs to be done to establish whether non-surgical treatment should be delivered with curative intent to these patients or not.

## Data Availability

The datasets used and/or analysed during the current study are available from the corresponding author on reasonable request.

## Abbreviations

CT: Computed tomography
CTV: Clinical target volume
dCRT: Definitive chemoradiotherapy
EC: Esophageal carcinoma
ESCC: Esophageal squamous cell carcinoma
EUS: Endoscopic ultrasound
FDG-PET: Fluorodeoxyglucose-positron emission tomography
GTV: Gross tumor volume
HR: Hazard ratio
LNs: Lymph nodes
OS: Overall survival
PTV: Planning target volume
RT: Radiotherapy
